# Anti-C5 monoclonal antibody treatment showing pathological resolution of complement-mediated atypical hemolytic uremic syndrome: a case report

**DOI:** 10.1186/s12882-024-03662-3

**Published:** 2024-07-15

**Authors:** Shigekazu Kurihara, Akinori Yamaguchi, Kosuke Sonoda, Yosuke Yamada, Makoto Harada, Koji Hashimoto, Hisashi Shimojo, Yoichiro Ikeda, Yuji Kamijo

**Affiliations:** 1 Department of Nephrology, Shinshu University School of Medicine, 3-1-1 Asahi, Matsumoto, Nagano, 390-8621 Japan; 2 Department of Pathology, Shinshu University School of Medicine, 3-1-1 Asahi, Matsumoto, 390-8621 Japan; 3 Division of Nephrology and Endocrinology, Graduate School of Medicine, University of Tokyo, 7-3-1 Hongo, Bunkyo-Ku, Tokyo, 113-8655 Japan

**Keywords:** Anti-C5 monoclonal antibody, Anti-CFH antibody, Atypical hemolytic uremic syndrome, Complement factor H mutation, Thrombotic microangiopathy, Pathological remission

## Abstract

**Background:**

No reports have shown histological changes before and after anti-C5 monoclonal antibody treatment in patients with atypical hemolytic uremic syndrome (aHUS). Here, we report a rare case of complement-mediated aHUS with a complement factor H (*CFH*) mutation and anti-CFH antibodies who underwent multiple kidney biopsies.

**Case presentation:**

A 53-year-old woman developed aHUS with *CFH* gene mutation [c.3572C > T (p. Ser1191 Leu)] and anti-CFH antibodies. Her father had succumbed to acute kidney injury (AKI) in his 30 s. She exhibited AKI, thrombocytopenia, and hemolytic anemia with schistocytes. After improving the platelet count with one session of plasma exchange, a kidney biopsy was performed one month after the onset of symptoms. Blood vessel thrombosis, obvious endothelial swelling, endocapillary hypercellularity, and subendothelial exudative lesions in the glomeruli and arterioles were detected. Anti-C5 monoclonal antibody treatment with eculizumab immediately improved disease activity. A second biopsy 3 months later revealed marked improvement of endothelial injuries with residual membrane double contours and exudative lesions. A third biopsy at 17 months after gradual improvement of kidney function showed a further decrease of double contours along with alterations of the exudative lesions to fibrous intimal thickening.

**Conclusions:**

This is the first report showing the pathophysiology of aHUS in the kidneys and the efficacy of anti-C5 monoclonal antibody treatment by presenting serial kidney pathological features before and after anti-C5 monoclonal antibody treatment. Since her *CFH* mutation was considered the most important pathological condition, treatment centered on eculizumab was administered, resulting in a good long-term prognosis. In addition, kidney pathological resolution in aHUS occurred over 1 year after anti-C5 monoclonal antibody treatment.

**Supplementary Information:**

The online version contains supplementary material available at 10.1186/s12882-024-03662-3.

## Background

Hemolytic uremic syndrome (HUS) is a form of thrombotic microangiopathy (TMA) mainly affecting the kidney and characterized by a triad of microangiopathic hemolytic anemia, thrombocytopenia, and consequent acute kidney injury (AKI) [1]. The International Hemolytic Uremic Syndrome group proposed the 2016 classification of HUS that included several disorders; shiga toxin-induced and pneumococcus-induced HUS, HUS associated with complement dysregulation or mutation of diacylglycerol kinase ε (DGKE), HUS related to cobalamin C defect, and HUS secondary to a heterogeneous group of causes (infections, drugs, cancer, and systemic diseases) [1]. Among them, complement-mediated aHUS is caused by uncontrolled activation of the alternative complement pathway at the endothelial cell surface [2]. Anti-C5 humanized monoclonal IgG antibody—including eculizumab and ravulizumab—recognizes complement protein C5 and blocks the terminal complement cascade [3, 4]. The Kidney Disease: Improving Global Outcomes (KDIGO) Controversies Conference proposed the treatment strategies for aHUS. In the strategies, a complement inhibitor treatment is considered as a first line treatment and is indicated for all patients diagnosed with primary aHUS [5]. However, no reports have compared the pathological kidney findings of a patient with complement-mediated aHUS before and after anti-C5 monoclonal antibody treatment. Here, we report a rare case of complement-mediated aHUS with a complement factor H (*CFH*) mutation and anti-CFH antibodies who underwent multiple kidney biopsies.

### Case Presentation

A 53-year-old woman was referred to our hospital by her family doctor after suffering from gastroenteritis for 2 weeks before admission (day 0, symptom onset date), followed by headaches, vomiting, and hypertension 1 week before admission. Her previous health checkup a year prior indicated normal kidney function. However, her father died of unexplained AKI in his thirties. Her height, body weight, and body mass index were 161 cm, 56.3 kg, and 21.6 kg/m^2^, respectively. Her blood pressure was high (174/85 mmHg), but other vital signs were normal. She had no abnormalities upon physical examination. Laboratory data at admission detected hemolytic anemia with schistocytes, thrombocytopenia, kidney dysfunction, urine abnormalities, and normal coagulation profile including ADAMTS13 activity. We observed that 50% hemolytic complement (CH50) activity was slightly high (57.1 U/mL, normal range 30–53 U/mL). Direct Coombs test, stool culture, pathogenic *Escherichia coli* immunosera tests, and ADAMTS13 inhibitor tests were negative (Table 1). Laboratory data and imaging detected no background diseases causing secondary TMA.

**Table 1:** Laboratory findings at admission

< Hematological findings >								
RBC	322 × 10^4^	/µL	ALP	183	IU/L	IC-C1q		( -)
Hb	9.9	g/dL	γGT	6	IU/L	IC-mRF		( -)
RET%	2.2	%	UN	45	mg/dL	ANA		( -)
Schistocytes	0.3	%	Cre	3.31	mg/dL	anti-cardiolipin Ab IgG		( -)
WBC	4,590	/µL	UA	8.5	mg/dL	anti-CL･β2GPI complex Ab		( -)
PLT	74,000	/µL	Na	141	mEq/L			
PT	11.7	sec	K	3.9	mEq/L	Lupus anticoagulant		( -)
APTT	25.4	sec	Cl	109	mEq/L	HIT Ab		( -)
Fibrinogen	340	mg/dL	Folic acid	5.4	ng/mL	PA-IgG		( -)
D-dimer	0.8	µg/mL	CRP	0.05	mg/dL	MPO-ANCA		( -)
TP	6.5	g/dL	CH50	57.1	mg/dL	PR3-ANCA		( -)
ALB	3.7	g/dL	C3	83	mg/dL	anti GBM Ab		( -)
Haptoglobin	< 5	mg/dL	C4	38.2	mg/dL	HIV Ag/Ab		( -)
IgG	1096	mg/dL	ADAMTS13 activity	70.3	%	HBs Ag		( -)
IgA	216	mg/dL				HCV Ab		( -)
IgM	114	mg/dL	ADAMTS13 inhibitor		( -)	PT IgG		( -)
T-Bil	1.53	mg/dL				FHA IgG		( -)
I-Bil	1.30	mg/dL	Direct Coombs test		( -)	VZV IgM		( -)
AST	21	IU/L				CMV antigenemia		( -)
ALT	9	IU/L	IC-C1q		( -)	ASO		( -)
LDH	669	IU/L	IC-mRF		( -)	ASK		( -)
< Urine findings >								
Protein	3 +		Sediment					
	3.6	g/gCre	U-RBC	50–99	/HPF	NAG	19.2	IU/L
Occult blood	3 +		Dysmorphic		( +)	β2-MG	28,900	µg/L
			Hyaline cast	100–999	/HPF			
			Granular cast	1–9	/HPF	Pneumococcus theca Ag		( -)

*RBC* red blood cells, *Hb* hemoglobin, *RET* reticulocyte, *WBC* white blood cells, *PLT* platelet, *PT* prothrombin time, *APTT* activated partial thromboplastin time, *TP* total protein, *ALB* albumin, *T-Bil* total bilirubin, *I-Bil* indirect bilirubin, *AST* aspartate aminotransferase, *ALT* alanine aminotransferase, *LDH* lactate dehydrogenase, *ALP* alkaline phosphatase, *γ-GT* gamma-glutamyl transferase, *BUN* blood urea nitrogen, *Cre* creatinine, *UA* uric acid, *Na* sodium, *K* potassium, *Cl* chloride, *CRP* C-reactive protein, *CH50* 50% hemolytic complement activity, *ADAMTS13* a disintegrin-like and metalloproteinase with thrombospondin type 1 motifs 13, *IC-mRF* immune complex, monoclonal rheumatoid factor assay, *ANA* anti-nuclear antibody, *Ab* antibody, *Ag* antigen, *HIT* heparin-induced thrombopenia, *PA-IgG* platelet associated immunoglobulin G, *MPO-ANCA* myeloperoxidase-anti-neutrophil cytoplasmic antibody, *PR3-ANCA* proteinase3-anti-neutrophil cytoplasmic antibody, *GBM* glomerular basement membrane, *PT* Pertussis Toxin, *FHA* Filamentous Hemagglutinin, *VZV* varicella-zoster virus, *CMV* cytomegalovirus, *ASO* anti-streptolysin O antibody, *ASK* anti-streptokinase antibody, *β2-MG* beta2-microglobulin, *NAG* N-acetyl-beta-glycosaminidase

**Fig. 1:**
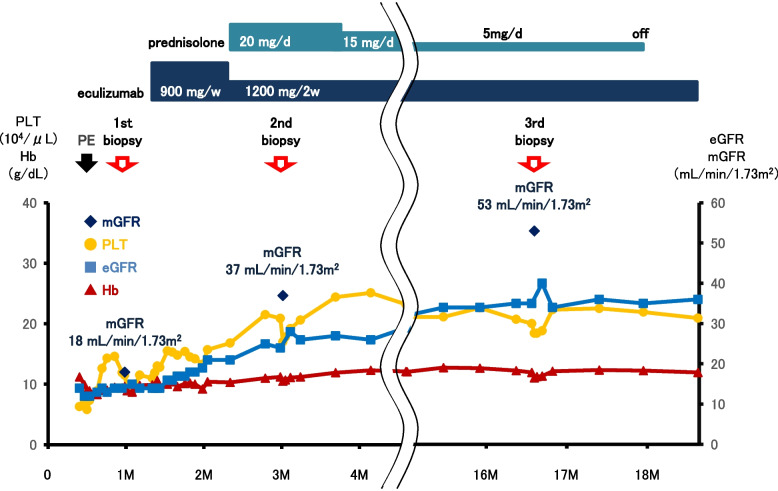
Clinical and therapeutic course. Eculizumab treatment improved kidney function, hemolytic anemia, and thrombocytopenia in a patient with aHUS who had a *CFH* mutation and anti-CFH antibodies. Blue, red, and yellow lines indicate the estimated glomerular filtration rate (eGFR) level, hemoglobin level, and platelet counts, respectively. Black rhombuses indicate the measured GFR (mGFR) level. Abbreviations: PE, plasma exchange; Hb, hemoglobin; PLT, platelet count

**Fig. 2:**
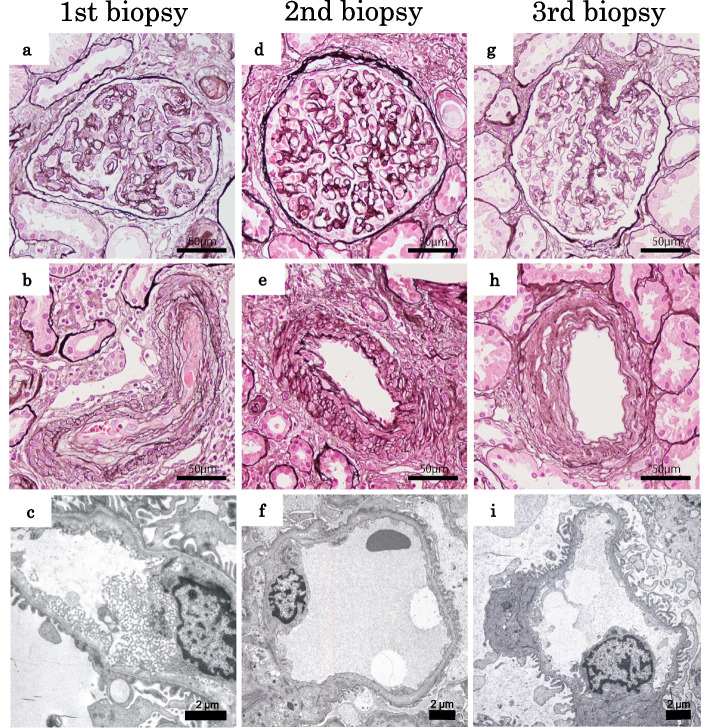
Pathological changes observed in kidney biopsies of a patient with aHUS pre- and post-eculizumab treatment. The upper and middle panels show representative light microscopic images of glomeruli and arterioles, respectively. Kidney biopsy specimens were stained with periodic acid-methenamine-silver. Scale bar = 50 µm. The lower panels show representative glomerular findings examined using an electron microscope. Scale bar = 2 μm. **a**–**c** First kidney biopsy findings before eculizumab treatment (1 month after symptom onset). Light microscopic analyses detected marked endothelial swelling, endocapillary proliferation, and subendothelial exudative lesions in both glomeruli and arterioles. GBM duplication and thrombosis in arterioles were also observed. Electron microscopy analysis detected subendothelial widening, endothelial cell swelling, and marked endothelial arcade formation. **d**–**f** Second kidney biopsy findings 3 months after symptom onset (2 months after eculizumab treatment). Acute injuries in glomeruli and arterioles (endothelial swelling, endocapillary proliferation and thrombosis, and endothelial arcade formation) had almost disappeared, but GBM duplication and subendothelial exudative lesions in arterioles remained. **g**–**i** Third kidney biopsy findings 17 months after symptom onset (16 months after the initial eculizumab treatment). GBM duplication was decreased, and glomeruli with minor abnormalities had increased. Subendothelial exudative lesions had almost disappeared in the arterioles, and fibrous intimal thickening had increased

At her next days of admission, 15 days after the onset of symptoms, her platelet count had decreased to 58,000 /µL, and one session of simple plasma exchange (PE) was performed 16 days after the onset of symptoms (Fig. 1). We calculated the patient’s plasma volume using her body weight and hematocrit value, and fresh frozen plasma equivalent to 1.1 times of her estimated plasma volume was used for PE. After one session of PE, the platelet count had recovered to a level that allowed a kidney biopsy (platelets 119,000/µL); however, severe kidney dysfunction persisted (serum creatinine level 2.89 mg/dL, estimated glomerular filtration rate [eGFR] 14 ml/min/1.73m^2^), and anemia did not improve (hemoglobin level 9.4 g/dL). Therefore, we performed the first kidney biopsy one month after the onset of symptoms. The kidney biopsy revealed thrombosis in blood vessels, glomerular endocapillary hypercellularity, swelling of endothelial cells, subendothelial exudative lesions in the glomeruli and arterioles, and glomerular basement membrane (GBM) duplication (Fig. 2a and b). Routine immunofluorescence analyses detected slight deposits of immunoglobulin (Ig)-A, IgM, C3c, and fibrinogen along glomerular capillaries, suggesting exudative changes (Supplemental Figure). Electron microscopy revealed subendothelial widening, endothelial cell swelling, and marked endothelial arcade formation, suggesting endothelial cell hypertrophy (Fig. 2c). The eGFR and measured glomerular filtration rate (mGFR) by inulin clearance showed severe kidney dysfunction (14 and 18 mL/min/1.73 m^2^, respectively). The pathological findings of TMA, the lack of findings of Shiga toxin-producing *Escherichia coli* infection-mediated HUS, thrombotic thrombocytopenic purpura, or secondary TMA, and the family history of AKI indicated complement-mediated aHUS. After confirming with a positive sheep erythrocyte hemolysis test, we started anti-C5 monoclonal antibody treatment (eculizumab 900 mg/week) 42 days after the onset of symptoms. Laboratory data improved immediately, and her CH50 activity dramatically decreased to below the detection limit (< 4 U/mL), suggesting complete blockage of the complement pathway. She was discharged on day 68. After 4 weeks of initial eculizumab treatment (900 mg/week), the dosage was changed to 1200 mg every 2 weeks. We detected a high titer of anti-CFH antibodies (112.2 AU/mL, normal range + 3SD using healthy control serum was 3.2 + 3.5 AU/mL) using an anti-CFH IgG ELISA kit (#KA1477, Abnova, Taipei City, Taiwan). To reduce her anti-CFH antibodies, glucocorticoids (prednisolone 20 mg/day) were administered from the 73rd day. We performed whole exome sequencing (WES) by next-generation sequencing (NGS) using the Agilent Sure Select V6 + UTR system. The following genes were analyzed: CFH, membrane cofactor protein (MCP), complement factor I (CFI), C3, complement component factor B (CFB), DGKE, thrombomodulin (THBD), and CFH-related protein (CFHR)1–5. This analysis detected two rare variants (minor allele frequency for both variants; MAF < 0.005), a known pathogenic mutation in *CFH* [c.3572C > T (p. Ser1191 Leu)] that causes complement-mediated aHUS, as well as benign/likely benign variant of complement factor I (*CFI*) [c.603A > C (p.Arg201Ser)].

After the introduction of eculizumab treatment, her kidney function began to gradually improved (Fig. 1). Three months after symptom onset, the patient’s eGFR and mGFR reached to 24 and 37 mL/min/1.73 m^2^, respectively; however, her severe kidney dysfunction persisted. She was concerned that her kidney function might not recover any further and requested a histological evaluation to examine the therapeutic effect of the anti-C5 monoclonal antibody treatment. After fully explaining the risks and benefits of a kidney biopsy and obtaining written informed consent, we performed a second kidney biopsy, which revealed scarce evidence of glomerular and arteriolar endothelial cell swelling, endocapillary hypercellularity, and arcade formation. However, GBM duplication and arteriolar subendothelial exudative lesions remained (Fig. 2 d–f). The proportion of glomerular endocapillary hypercellularity decreased from 57 to 16% and GBM duplication decreased from 81 to 66% (Table 2). Minor glomerular abnormality increased slightly from 14 to 22%. In the arterioles, endothelial proliferation decreased from 37 to 0%, and subendothelial exudative lesions decreased from 37 to 21%. Given these improved pathological findings, glucocorticoid treatment was gradually tapered. We reevaluated the anti-CFH antibodies 5 months after symptom onset and found that her anti-CFH antibody titer did not change despite steroid therapy (104.8 AU/mL). However, her CH50 activity remained below the detection limit, suggesting that her complement pathway was completely blocked by the anti-C5 monoclonal antibody treatment. TMA biological parameters and pathological findings were markedly improved despite the presence of anti-CFH antibodies, and the *CFH* pathogenic mutation necessitated the continuation of anti-C5 monoclonal antibody treatment. Considering these results, we decided that steroid therapy and other immunosuppressive treatments aimed at reducing the anti-CFH antibodies were not important clinically. Therefore, we tapered steroid treatment further, and continued anti-C5 monoclonal antibody treatment without using other immunosuppressive treatments. Continued eculizumab treatment further improved the patient’s kidney function, and 13 months after symptom onset, her eGFR levels were approximately 34 ± 2 mL/min/1.73 m^2^. Since tissue repair was not complete at her second kidney biopsy, the patient expected further improvement in kidney function. However, her kidney function was slowly recovering, but did not return to the normal level as she hoped. Therefore, she requested pathological reevaluation again. After informed consent concerning the risks of kidney biopsy, a third kidney biopsy was conducted 17 months after symptom onset. Her eGFR and mGFR were 35 and 53 mL/min/1.73 m^2^, respectively. The remaining GBM duplication had decreased to 36%, and minor glomerular abnormality increased to 58% (Fig. 2 g, and Table 2). In the arterioles, the remaining subendothelial exudative lesions had almost disappeared, and fibrous intimal thickening was increased (Fig. 2 h and Table 2). We considered that the kidney lesions had reached remission level pathologically, and glucocorticoids were discontinued 18 months after symptom onset. We continuously monitored her CH50 activity throughout her clinical course to help judge whether there was complete blockage of the complement pathway. Her CH50 activity persisted below the detection limit, and we tried to extend the eculizumab treatment with a dosing interval of 1200 mg every 3 weeks after 19 months and then 1200 mg every 4 weeks after 34 months. Despite the extension of the treatment interval, non-detection of CH50 activity remained, and her eGFR levels gradually increased and reached a plateau at 42 ± 2 mL/min/1.73 m^2^. At 76 months from the onset of symptoms, no recurrence of TMA was observed. We will continue anti-C5 monoclonal antibody treatment to prevent TMA recurrence.

**Table 2:** Summary of kidney biopsy findings

	1st biopsy	2nd biopsy	3rd biopsy
Total number of obtained glomeruli	60	48	42
Number of global scleroses	23	16	6
Number of assessment glomeruli (Total glomeruli) – (global sclerosis)	37	32	36
Number of glomeruli with endocapillary hypercellularity (EH)	21	5	5
Proportion of endocapillary hypercellularity Glomeruli with EH/assessment glomeruli (%)	57%	16%	14%
Number of glomeruli with glomerular basement membrane (GBM) duplication (GBMD)	30	21	13
Proportion of GBM duplication Glomeruli with GBMD / assessment glomeruli (%)	81%	66%	36%
Number of glomeruli with minor glomerular abnormality (MGA)	5	7	21
Proportion of minor glomerular abnormality Glomeruli with MGA / assessment glomeruli (%)	14%	22%	58%
Number of assessment arterioles	30	24	27
Number of arterioles with endothelial proliferation (EP)	11	0	0
Proportion of endothelial proliferation Arterioles with EP/assessment arterioles (%)	37%	0%	0%
Number of arterioles with subendothelial exudative lesions (SEL)	11	5	1
Proportion of subendothelial exudative lesions Arterioles with SEL / assessment arterioles (%)	37%	21%	4%
Number of arterioles with fibrous intimal thickening (FIT)	3	4	6
Proportion of fibrous intimal thickening Arterioles with FIT/assessment arterioles (%)	10%	17%	22%

## Discussion and conclusions

**Fig. 3:**
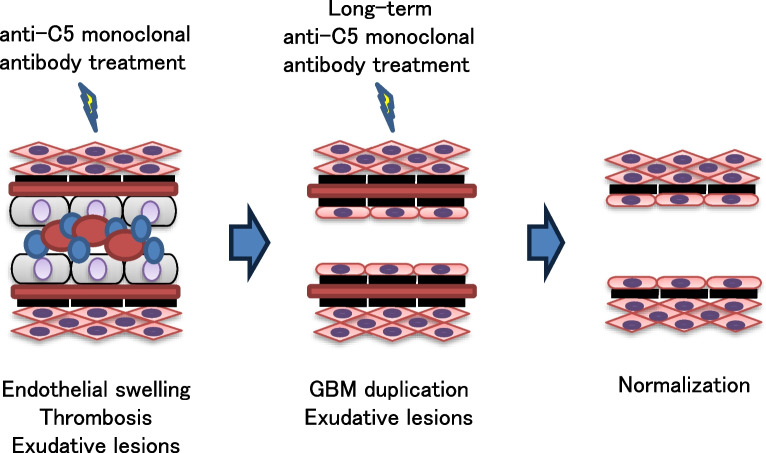
Schema of speculative pathological changes in the aHUS kidney due to anti-C5 monoclonal antibody treatment. In the early stages, kidney function improves quickly with a reduction of thrombosis, endocapillary hypercellularity, and endothelial swelling in the glomeruli and arterioles. However, GBM duplication and subendothelial exudative lesions may remain. Continued anti-C5 monoclonal antibody treatment helps to gradually repair these remaining lesions

We describe the pathological repair process in the kidney of a patient with complement-mediated aHUS through a series of kidney biopsies before and after anti-C5 monoclonal antibody treatment. Obtaining pathological images before treatment is difficult because of the severe bleeding risk in patients with aHUS. Udagawa et al. reported a case where two kidney biopsies were obtained after eculizumab treatment [6]. However, post-treatment pathology would be considerably modified. In our case, the patient’s platelet count was improved by single plasmapheresis so that a kidney biopsy could be performed prior to anti-C5 monoclonal antibody treatment. A single session of plasmapheresis may affect kidney pathology. However, we thought the effects would be minor. This report presents the speculated kidney repair process—following aHUS-related damage—throughout anti-C5 monoclonal antibody treatment (Fig. 3). In the early stages, kidney function improves quickly with a reduction of thrombosis, endocapillary hypercellularity, and endothelial swelling in the glomeruli and arterioles. However, GBM duplication and subendothelial exudative lesions may remain. Continued anti-C5 monoclonal antibody treatment helps to gradually repair these remaining lesions and kidney function continues to slowly improve. This is the first report showing the pathophysiology of aHUS in kidneys and the efficacy of anti-C5 monoclonal antibody treatment by presenting serial pathological features of the kidney before and after anti-C5 monoclonal antibody treatment. This case report supports the hypothesis that kidney pathological resolution in complement-mediated aHUS requires a long period of over 1 year after initiating anti-C5 monoclonal antibody treatment.

Most complement-mediated aHUS cases are caused by a genetic mutation in the complement cascade or by antibodies to complement-related proteins. In Japan, the percentage of complement-mediated aHUS cases caused by C3 gene mutation, CFH mutations, and anti-CFH antibodies are 27%, 8%, and 16%, respectively [7]. Anti-CFH antibodies induce functional CFH deficiency by binding to its C-terminal region and reducing its regulatory function [8]. It was reported that anti-CFH antibodies was associated with various variants of CFHRs such as homozygous deficiency of *CFHR1* and homozygous deletion of *CFHR3*-*CFHR1* [8–11]. These *CFHR* variations cause deficiency of CFHR proteins and CFH autoantibody positive (DEAP)-HUS [9]. Considering the possibility of association of CFHR variants in this patient, we sequenced *CFHR1-5*, but no non-synonymous single nucleotide polymorphisms or insertion-deletions were detected in *CFHR1-5*. However, we did not perform multiplex ligation-dependent probe amplification to investigate the presence of the CFHRs copy number variation typically linked with the anti-CFH antibodies. The mechanism leading to the presence of anti-CFH antibodies in this patient is unknown; thus, more detailed genetic analysis is needed in future.

In addition to anti-CFH antibodies, this case has the variant of *CFH* [c.3572C > T (p. Ser1191 Leu)], a well-known pathogenic mutation that causes aHUS [12]. Factor H is composed of 20 short consensus repeat (SCR) domains [13]. Two major functional regions are located at the N- and C-terminal of the CFH molecule. The N-terminal domains of SCRs 1–4 mediate the complement regulatory activities of CFH, and the C-terminal domains of SCRs 19–20 allow the attachment of CFH to host cells and inhibit complement activation at the cell surface [13]. SCRs 16–20 are reported to be the principal CFH domain involved in the pathogenies of aHUS [14]. The variant of *CFH* [c.3572C > T (p. Ser1191 Leu)] results in a non-conservative amino acid change located in the C-terminal domain of SCR20 of the encoded protein sequence [15]. Since SCR20 is known to be a very important hot spot for mutations in the familial aHUS [16], this variant is undoubtedly one of the major causes of aHUS in this case. Since the patient had two important pathogenic factors, anti-CFH antibodies and *CFH* mutation, which both inhibit the C-terminal function of CFH, both factors are likely to exert substantial impact on the progression of aHUS.

Treatment options for complement-mediated aHUS include plasma therapy, anti-complement therapy, and immunosuppressants and corticosteroid for suppression of autoantibody production [17]. Until 2011, the main therapy for complement-mediated aHUS was plasma therapy including plasma infusion and PE. Reports on the efficacy of plasma therapy are inconsistent and controversial, however plasma therapy is the only therapy with near complete global availability and therefore it remains one of the important treatments for complement-mediated aHUS [17]. Today, however, the first line of treatment in complement-mediated aHUS is anti-complement therapy, with anti-C5 antibody. The KDIGO Controversies Conference stated that all patients with a clinical diagnosis of primary aHUS are eligible for treatment with a complement inhibitor [5]. Clinical evidence suggests the importance of earlier clinical intervention with complement inhibitor. When PE is prioritized despite the availability of anti-C5 antibodies, introduction of eculizumab treatment may be delayed, and may lead to a suboptimal therapeutic outcome. In the treatment for anti-CFH antibodies-mediated aHUS, removal of the antibodies using PE and/or immune suppression therapies using corticosteroid, cyclophosphamide, mycophenolate mofetil, or rituximab is important [17]. Various combinations of PE and/or immunosuppressive therapies have been tried and good outcomes reported, but there is no standard treatment protocol [17–19]. There is no clear evidence to show whether antibody removal therapy or anti-complement therapy is more effective, however preliminary data suggest that anti-C5 antibodies are also effective in anti-CFH antibodies-mediated aHUS [17]. On the basis of these suggestions, we need to consider the optimal treatment in our case, having both *CFH* mutation and anti-CFH antibodies. The prognosis of a person with a *CFH* mutation is generally worse than that of a person with anti-CFH antibodies [20, 21] and our patient’s *CFH* mutation occurs in the last SCR20 with high susceptibility to aHUS, suggesting poor prognosis without anti-complement therapy. Her *CFH* mutation was considered to be the most important pathological condition, and so treatment centered on eculizumab was administered, resulting in a good long-term prognosis. Since our case is very rare, it is unclear whether our observed therapeutic effects and clinical course can be generalized. The genetic backgrounds that cause aHUS are diverse and the therapeutic response is reported to differ [22, 23], suggesting the need to tailor the therapy of patients with aHUS on a case-by-case basis.

The recurrence rate of aHUS after discontinuation of eculizumab varies depending on the cause of aHUS. In a report of 38 patients with aHUS who discontinued eculizumab, 12 patients (31%) experienced recurrence of aHUS during a median observation period of 22 months. Eight of 11 patients (72%) with *CFH* mutations and four of eight patients (50%) with MCP mutations experienced recurrence; however, no recurrence was observed in the 16 patients who did not have the genetic mutation that causes aHUS, suggesting a very high recurrence rate in patients with aHUS due to *CFH* mutation [24]. Therefore, we continued eculizumab treatment in our patient. Eculizumab has been administered once every 2 weeks; however, we suggest that an administration interval extension to 4 weeks is possible under careful monitoring of CH50 activity. Of note, a CH50 activity assay can screen the residual functional activity of C5 and determine the efficacy of anti-C5 monoclonal antibodies [25]. Volokhina et al. reported that eculizumab treatment completely blocked the complement pathway for 4 weeks [26], and therapeutic range concentrations are maintained with administration every 3 or 4 weeks [27]. Anti-C5 monoclonal antibody treatment is very expensive, and reducing the burden on the medical economy is important. Therefore, evidence supporting the possibility of extending the administration interval of anti-C5 monoclonal antibodies with CH50 activity monitoring is valuable. A larger-scale cohort study is needed to explore the efficacy of eculizumab interval extension. Recently, the long-acting anti-C5 humanized monoclonal antibody ravulizumab was approved for the treatment of aHUS. It is reported that treatment with ravulizumab administered every 8 weeks in patients with aHUS who were previously treated with eculizumab resulted in stable kidney and hematologic parameters with no unexpected safety concerns [28]. When this long-acting C5 inhibitor is used, it may be possible to further extend the treatment interval with CH50 activity monitoring and improve the patient quality of life; therefore, we are considering switching from eculizumab to ravulizumab in the near future.

aHUS is a TMA, the pathological features of which represent tissue responses to endothelial injury, however kidney pathology cannot determine etiology [5]. Moreover, the improvement of histological lesions is expected based on the improvement of TMA biological parameters, and repeated kidney biopsies may be considered unnecessary. Although serological parameters are useful in predicting improvement in disease status, the sensitivity of serological parameters is not necessarily high, and histological changes are more associated with prognosis. For example, repeated protocol kidney biopsy is common in kidney transplantation management, as it can predict various events such as subclinical rejection, chronic allograft nephropathy, and graft dysfunction due to nonimmune factors [29]. These events cannot be determined by serological data alone. Furthermore, repeated kidney biopsy has uncovered surprising phenomena, including a reversal of diabetes mellitus nephropathy after pancreatic transplantation [30]. In TMA management, the time-dependent relationship between TMA biological parameters and pathological changes has not been widely reported, and there is no evidence that improvement in TMA biological parameters is linked to pathological changes. We believe that this report is of great clinical importance, because it clarifies the long-term gradual kidney repair process of complement-mediated aHUS after anti-C5 monoclonal antibody treatment regardless of the quick improvement of TMA biological markers. As kidney biopsy has the risk of causing adverse events, such as bleeding, the risks and benefits should be explained to the patient prior to the procedure and the patient should provide written informed consent.

### Supplementary Information

Additional file 1. Routine immunofluorescence findings.Fresh frozen kidney specimens were used for routine immunofluorescence analyses for IgA, IgG, IgM, C3c, and fibrinogen. First kidney biopsy findings before eculizumab treatment (1 month after symptom onset) detected slight deposits of IgA, IgM, C3c, and fibrinogen along glomerular capillaries, suggesting exudative changes. After eculizumab treatment, these deposits gradually disappeared.

## Data Availability

The data used in this study are available from the corresponding author upon request.

## Abbreviations

AKI: Acute kidney injury
aHUS: Atypical hemolytic uremic syndrome
CFH: Complement factor H
DEAP: Deficiency of CFHR proteins and CFH autoantibody positive
eGFR: Estimated glomerular filtration rate
GBM: Glomerular basement membrane
HUS: Hemolytic uremic syndrome
KDIGO: Kidney Disease: Improving Global Outcomes
mGFR: Measured glomerular filtration rate
NGS: Next-generation sequencing
PE: Plasma exchange
SCR: Short consensus repeat
TMA: Thrombotic microangiopathy
WES: Whole exome sequencing
Ig: Immunoglobulin
