# Supplementary figures and images for: Anti-C5 monoclonal antibody treatment showing pathological resolution of complement-mediated atypical hemolytic uremic syndrome: a case report
Source: BMC Nephrol. 2024 Jul 15;25:224. doi: 10.1186/s12882-024-03662-3 (PMC11247795; doi:10.1186/s12882-024-03662-3)

## Slide 1
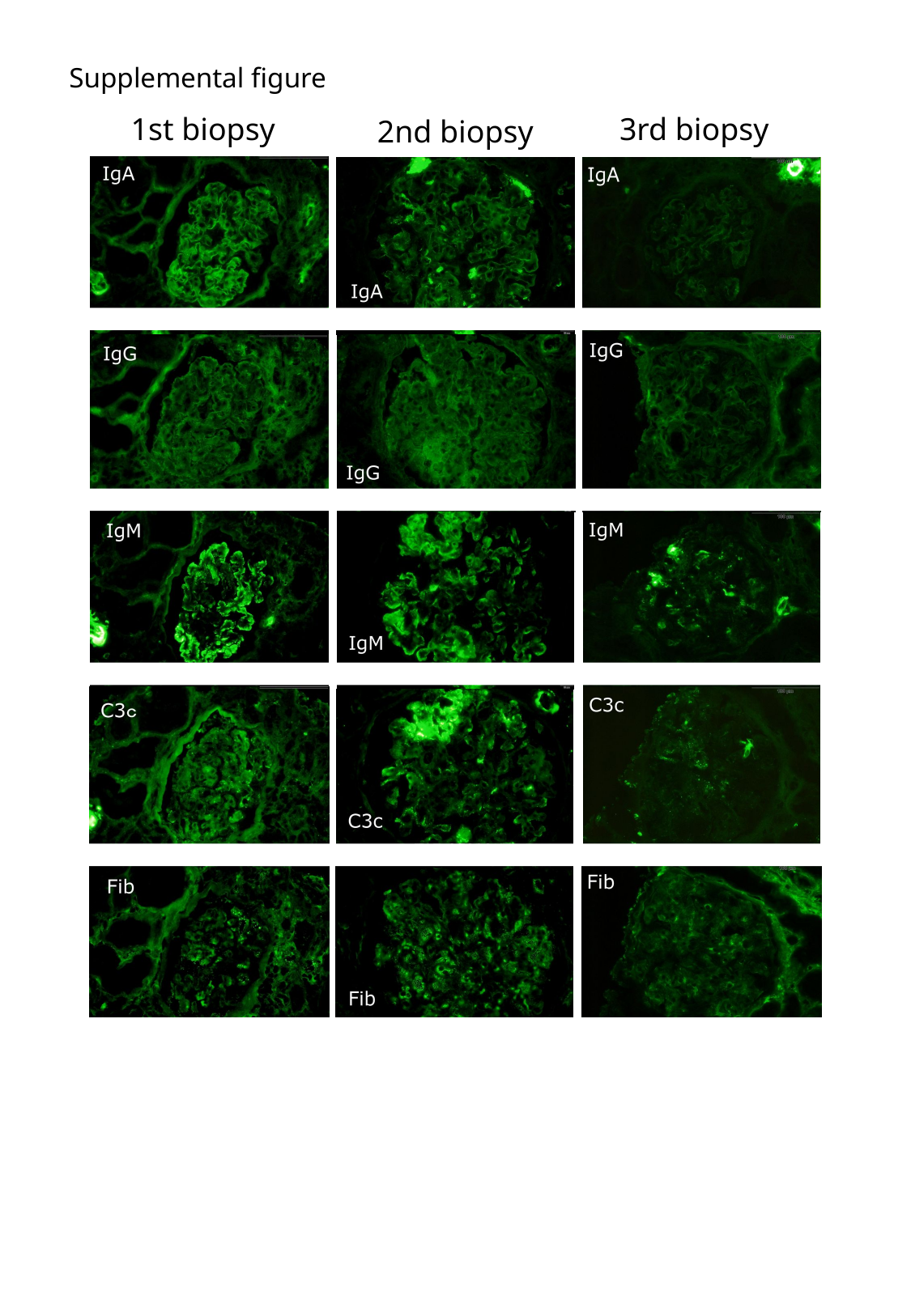

Supplemental figure
1st biopsy
3rd biopsy
2nd biopsy

Supplement: Supplementary file 1 — Additional file 1. Routine immunofluorescence findings.Fresh frozen kidney specimens were used for routine immunofluorescence analyses for IgA, IgG, IgM, C3c, and fibrinogen. First kidney biopsy findings before eculizumab treatment (1 month after symptom onset) detected slight deposits of IgA, IgM, C3c, and fibrinogen along glomerular capillaries, suggesting exudative changes. After eculizumab treatment, these deposits gradually disappeared. [file 12882_2024_3662_MOESM1_ESM.pptx]
